# Hepatoprotective Effect of Jianpi Huoxue Formula on Nonalcoholic Fatty Liver Disease Induced by Methionine-Choline-Deficient Diet in Rat

**DOI:** 10.1155/2019/7465272

**Published:** 2019-07-01

**Authors:** Yu Feng, Yan Chen, Binrui Yang, Qingping Lan, Tao Wang, Guozhen Cui, Zhitao Ren, I. Cheong Choi, George Pak-Heng Leung, Fenggen Yan, Dacan Chen, Hon Ho Yu, Simon Ming Yuen Lee

**Affiliations:** ^1^State Key Laboratory of Quality Research in Chinese Medicine and Institute of Chinese Medical Sciences, University of Macau, Macau; ^2^Department of Bioengineering, Zhuhai Campus of Zunyi Medical University, Zhuhai, China; ^3^Department of Gastroenterology, Kiang Wu Hospital, Macau; ^4^Department of Pharmacology and Pharmacy, The University of Hong Kong, Hong Kong; ^5^The Second Affiliated Hospital of Guangzhou University of Chinese Medicine, Guangdong Provincial Hospital of Chinese Medicine, Guangzhou, Guangdong, China

## Abstract

In parallel with the prevalence metabolic syndrome, nonalcoholic fatty liver disease (NAFLD) has become the most common chronic liver disease in most countries. It features a constellation of simple steatosis, nonalcoholic steatohepatitis (NASH), fibrosis, cirrhosis, and even hepatocellular carcinoma. There are no approved drugs for effective management of NAFLD and NASH. Jianpi Huoxue formula (JPHX) mainly consists of Atractylodes macrocephal* (Baizhu)*, Salvia miltiorrhiza (*Danshen*), Rasux Paeonia Alba (*Baishao*), Rhizoma Alismatis (*Zexie*), and Fructus Schisandrae Chinensis (*Wuweizi*), which may have beneficial effects on NAFLD. The aim of the study was to identify the effect of JPHX on NAFLD. A NAFLD model was induced by methionine-choline-deficient food (MCD) in Wistar rats and orally administered with simultaneous JPHX, once a day for 8 weeks. Hepatocellular injury, lipid profile, inflammation, fibrosis, and apoptosis were evaluated. The results showed that JPHX significantly decreased the abnormal serum alanine aminotransferase (ALT) and aspartate aminotransferase (AST) levels compared with the MCD model (P<0.05). Furthermore, JPHX protected MCD diet-fed rats from accumulation of hepatic triglycerides (TG) and total cholesterol (TC). Histological examination demonstrated that JPHX noticeably normalized the NAFLD activity score (NAS). Moreover, JPHX ameliorated liver inflammation by decreasing TNF-*α* levels and reduced collagen and matrix metalloproteinases in MCD diet-fed rats. In addition, JPHX prevented rats from MCD-induced cellular apoptosis, as suggested by TUNEL staining, and suppressed the activation of caspase 3 and 7 proteins. JPHX also inhibited the phosphorylation of JNK. In conclusion, JPHX exhibited a hepatoprotective effect against NAFLD in an MCD experimental model.

## 1. Introduction

Nonalcoholic fatty liver disease (NAFLD) is the most common chronic liver disease in most countries and regions including the United States, Asia, the Middle East, and Europe [1]. The prevalence of NAFLD worldwide has been estimated at 25.24% and it shows an increased trend with the high prevalence of obesity, diabetes, hyperlipidemia, and metabolic syndrome [2–4]. NAFLD can be divided into two major subtypes: simple steatosis and nonalcoholic steatohepatitis (NASH). Most patients do not progress to cirrhosis, which is mainly characterized by steatosis especially macrovascular steatosis, while almost 20% patients will have a progressive liver disease called NASH [5]. Patients with NASH usually develop liver steatosis, more severe lobular and portal inflammation, and ballooning and have a higher chance of developing fibrosis, cirrhosis, and hepatocellular carcinoma [6]. However, currently, there are no approved drugs for effective management of NAFLD and NASH, which have become a major global public health problem and place an enormous burden on health-care systems and societies. Some preventive strategies, such as lifestyle modification and an energy‐restricted diet, have achieved limited success including improved steatosis and decreased aberrant aminotransferases [7]. Therefore, it is critical to develop new therapeutic targets for NAFLD treatment. Nowadays, many researches are giving new hope to patients with NAFLD and those at high risk of developing NAFLD.

In Chinese medicine philosophy, the pathogenesis of NAFLD includes spleen vacuity, liver stagnation, and phlegm-damp obstruction. Thus far, few traditional Chinese medications have been studied for treatment of patients with NAFLD [8–10]. Jianpi Huoxue (JPHX), as a Chinese herbal formula, has been used for liver disease in China for many years [11]. JPHX mainly consists of Atractylodes macrocephal* (Baishu)*, Salvia miltiorrhiza (*Danshen*), Rasux Paeonia Alba (*Baishao*), Rhizoma Alismatis (*Zexie*), and Fructus Schisandrae Chinensis (*Wuweizi*) and strengthens the spleen, emolliates the liver, dispels dampness, dissolves stasis, and promotes blood circulation [12, 13]. JPHX contains active compounds that may regulate lipid metabolism and exhibit anti-inflammatory properties. The aim of the study was to determine the role of JPHX in the development of MCD diet-induced NAFLD in rats.

We hypothesized that JPHX may have a hepatoprotective effect during progression of NAFLD. Clinically, NAFLD is characterized by elevated serum aminotransferases, accumulation of fat with more than 5% hepatocytes and no history of alcohol abuse [1, 5, 14]. Animal models of NAFLD can be induced in rodents, as with many other human diseases, by common dietary protocols, and used for developing new therapeutic approaches and investigating the disease mechanisms [15]. MCD diet has been used for over 40 years to induce NAFLD, as a classic model that showed hepatic histological features including steatosis, inflammation, and fibrosis in a short time [16]. In the present study, MCD diet-induced NAFLD in rat was used to explore JPHX as a therapeutic agent for NAFLD.

## 2. Materials and Methods

### 2.1. Animal Models

Male Wistar rats, weighing approximately 220g, were fed a methionine- and choline-deficient (MCD diet) (Trophic Animal Feed High-Tech Co., Ltd., Nantong, China) that contained amino acids, corn oil, fibers, vitamins, minerals, sodium bicarbonate, and tertiary butylhydroquinone (TBHQ), or the same diet supplemented with methionine and choline (MCS diet) for up to 8 weeks. The substrain of Wistar rats is Wistar IGS rats, which are outbred. All rats were randomly divided into five groups (6-9 rats per experimental group) including an MCS group, MCD group, and JPHX 0.60 g/kg, JPHX 1.21 g/kg, and JPHX 2.42 g/kg groups. Rats were housed in groups of 2~4 in IVC cages with comfortable bedding, and the animal room with a 12-hour dark/light cycle temperature ranged between 21 and 25°C; humidity was maintained between 55% to 65%. All rats had free access to food and water and were weighed at weekly intervals.

### 2.2. JPHX Formula

JPHX formula was provided by Shanghai Sunrise Traditional Chinese Medicine Co., Ltd., Shanghai, China. The NAFLD model was induced by MCD diet in rats, orally administered simultaneously with JPHX once a day until rats were sacrificed. JPHX formula has a long history of use in clinical practice in China, therefore the different JPHX doses were chosen according to previous clinical experiences. The rat dose was converted from human dose which was multiplied by 7 based on the body surface area. A dose-response study is a valid research design to evaluate the efficacy of TCM [17–19]. Three doses of JPHX formula were administrated to the rats. Rats in JPHX treatment group were administered with JPHX at three different doses of 0.60, 1.21, or 2.42 g/kg (weight of rat). Powdered formula (10.00 g) was dissolved in 50 mL distilled water and then the rats were orally administered with JPHX solution according to their body weight. The MCS group and MCD vehicle group were orally treated with distilled water. The animal study conformed to the ethical standards (UMARE-001-2017) of the Institute of Chinese Medical Science, University of Macau.

### 2.3. Determination of Serum and Liver Biochemical Parameters

Blood samples were collected every 2 weeks from the tale vein and 1-2 mL of blood can be obtained and centrifuged at 1000 r.c.f for 15 min at 4°C. Serum was separated to detect biomarkers, including alanine aminotransferase (ALT), aspartate aminotransferase (AST), triglyceride (TG), and total cholesterol (TC) (Nanjing Jiancheng Biotech Co., Nanjing, China). Tumor necrosis factor-*α* (TNF-*α*) levels were measured by an ELISA kit (Duo-Set; R&D Systems Inc., MN, Minnesota, USA), and absorbance was read at 450 nm by a microplate reader according to the manufacturer's instructions. At the end of 8 weeks, blood samples from abdominal aorta were collected and approximately 5 mL of blood can be collected to be analyzed; hepatic TG and TC levels were determined by the same kit used for serum, as previously described [20]. Briefly, lipid was extracted using chloroform/methanol solution. The organic phase was collected and then vaporized, and the dry powder obtained was dissolved in isopropanol for the detection of hepatic TG and TC. Hepatic glutathione peroxidase (GPx) activity was detected to analyze oxidative stress by commercial kit from Nanjing Jiancheng Biotech Co. (Nanjing, China) [21].

### 2.4. Histopathological Analysis

At the end of 8 weeks, liver samples were collected. Livers were washed with ice PBS, dried with filter paper, and weighed. A part of the fresh liver tissue was fixed in 4% paraformaldehyde, embedded in paraffin, cut into 6-*μ*m-thick slices, and stained with hematoxylin and eosin (H&E) and Sirius red stain for histopathology analysis. Histology was assessed in a blind manner by a pathologist. Also, a part of the fresh liver tissue was embedded in optimal cutting temperature compound (OCT) for staining with Oil red O (Sigma, St. Louis, MO, USA). Terminal deoxynucleotidyl transferase (TdT)-mediated dUTP nick-end (TUNEL) assay was performed for apoptosis using a commercial kit according to the manufacturer's instructions (Beyotime Institute of Biotechnology, Nantong, China). Quantification of apoptosis was performed by counting the number of TUNEL-positive cells in three random fields (20 × magnification). Expression of *α*-SMA was determined using immunohistochemistry according to the standard method; paraffin-embedded sections were immunostained with an *α*-SMA antibody [22].

### 2.5. Quantitative Real-Time Polymerase Chain Reaction Analysis (qRT-PCR)

Fresh liver samples were collected and snap-frozen in liquid nitrogen and then stored at -80°C for further analysis. Total RNA was extracted from hepatic tissue with TRIzol reagent (Invitrogen, Carlsbad, CA, USA) and reverse-transcribed into cDNA using SuperScript reverse system (Invitrogen) for amplification according to the manufacturer's protocol. Quantitative real-time polymerase chain reaction (RT-PCR) was performed by SYBR* Premix Ex Taq*™ II (Tli RNaseH Plus) (Takara, Shiga, Japan). Primers information was listed in Table 1.

### 2.6. Western Blotting

Liver tissue from different groups was homogenized with stainless steel beads in RIPA lysis buffer by TissueLyser II (QIAGEN, Hilden, Germany) and then centrifuged at 12,500 r.c.f for 30 min at 4°C. Supernatant was obtained and protein concentrations were determined by a Pierce BCA protein assay kit (Thermo Fisher Scientific, Waltham, MA, USA). Equal amounts of protein and molecular weight markers were loaded on sodium dodecyl sulfonate polyacrylamide gel electrophoresis (SDS-PAGE). Protein samples were transferred to a polyvinylidene difluoride (PVDF) membrane that was blocked with 5% nonfat milk in 1× Tris-buffered saline with 0.5% Tween-20, for 1 hour at room temperature. Membranes were incubated with primary antibodies overnight at 4°C. Horseradish peroxidase (HRP)-linked secondary antibodies (Cell Signaling Technology, Danvers, MA, USA) were incubated at room temperature for 1 hour after washing at least three times with Tris-buffered saline Tween 20 (TBST), for 10 min each time. Signals were detected by ECL detection kit (GE Healthcare, Milwaukee, WI, USA) and quantified with Image Lab software (Bio-Rad, Hercules, CA, USA).

### 2.7. Data Analysis and Statistics

All data were collected from three independent experiments. Student t-test was used to compare differences between MCS group and MCD group. Differences among experimental groups were evaluated by one-way ANOVA, followed by Dunnett's multiple comparisons test. P values less than 0.05 were regarded as statistically significant. Data are described as mean ± standard deviations (SD). Alpha level was two tailed. The GraphPad prism was used for statistical analysis (version 6.0, GraphPad Inc., San Diego, CA, United States).

## 3. Results

### 3.1. JPHX Does Not Influence Body Weight and the Liver-to-Body Weight Ratio (LBW) in NAFLD

**Figure 1:**
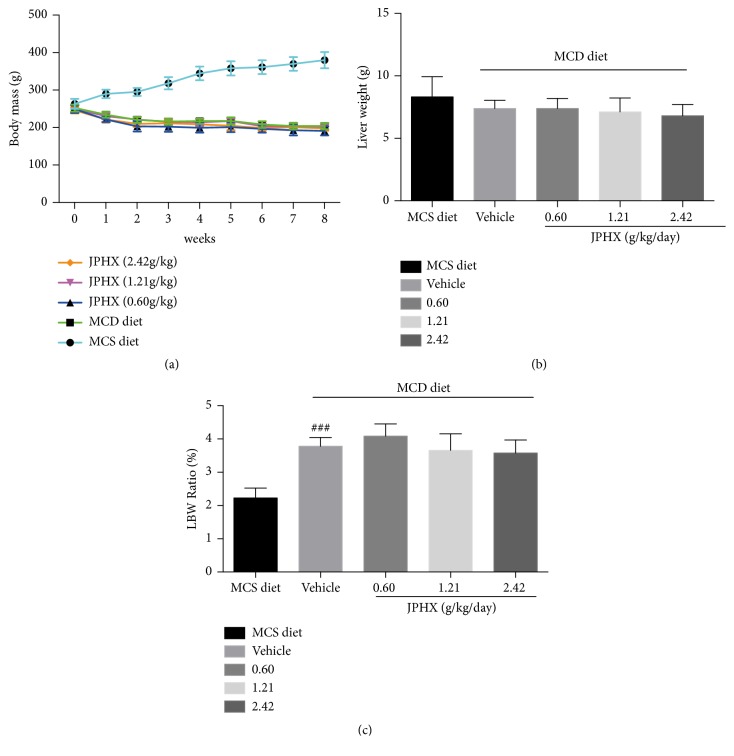
Effect of MCD diets on body weights, liver weights, and liver/body weight ratio in rats. (a) Body weight of rats. (b) Liver weight of rats after 8 weeks of the feeding. (c) Liver/body weight ratio of rats at 8 weeks. Values are shown as the mean ± SD. MCS n=6, MCD n=9, and JPHX treatment groups n=7. #*p*<0.05, ##*p*<0.01, and ###*p*<0.001, MCD diet vehicle group versus MCS diet group. *∗p*<0.05, *∗∗p*<0.01, and *∗∗∗p*<0.001, MCD diet vehicle group versus JPHX treatment groups.

In our study, JPHX was performed on rats using an MCD diet-induced NAFLD model. The body weights of MCS diet-fed rats showed an increasing trend, while the body weights of MCD diet-fed rats gradually decreased, by approximately 20%-30%, during the 8 weeks of testing period because of a noticeably lower caloric intake (Figure 1(a)). The liver weights of the MCD vehicle group and JPHX treatment group were similar (Figure 1(b),* p* = 0.1608). In addition, the liver/body weight ratio (LBW) in MCD diet-fed rats was higher than that in MCS diet-fed rats (Figure 1(c),* p* < 0.0001). We observed that JPHX formula therapy did not significantly influence the body weights and LBW of rats.

### 3.2. JPHX Inhibits Hepatic Steatosis

**Figure 2:**
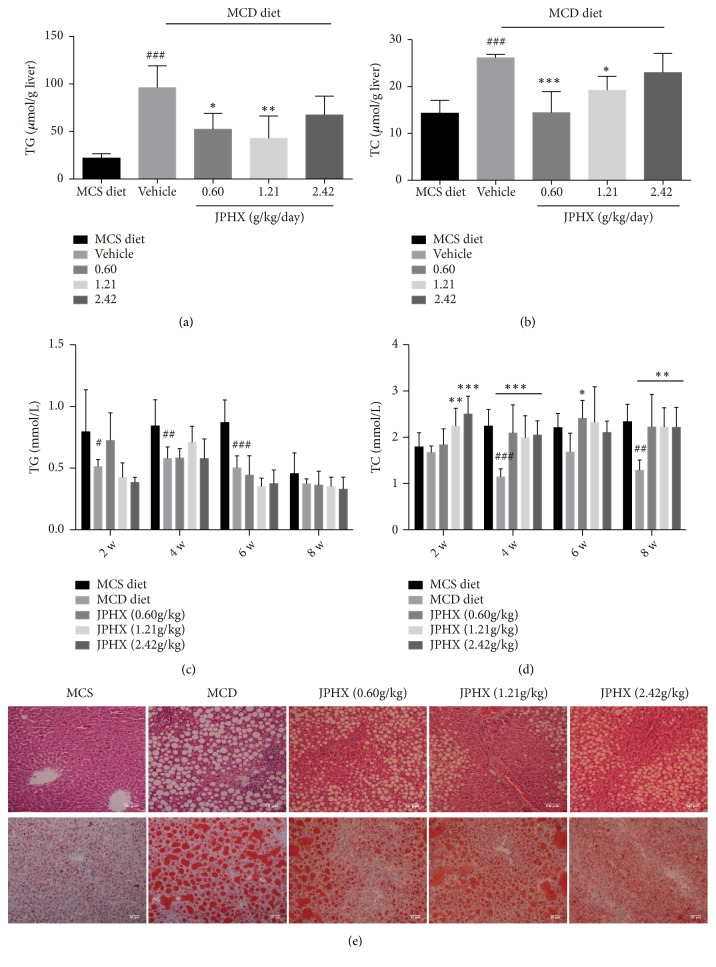
Effect of JPHX on accumulation of hepatic TG and TC induced by MCD diet and liver histology. (a) Liver TG levels. (b) Liver TC levels. (c) Serum TG levels. (d) Serum TC levels. (e) H&E and Oil red O staining of representative liver sections from each treatment group. Original magnification × 200. Values are shown as the mean ± SD. MCS n=6, MCD n=9, and JPHX treatment groups n=7. #*p*<0.05, ##*p*<0.01, and ###*p*<0.001, MCD diet vehicle group versus MCS diet group. *∗p*<0.05, *∗∗p*<0.01, and *∗∗∗p*<0.001, MCD diet vehicle group versus JPHX treatment groups.

Serum and liver TG and TC levels were detected to assess the effect of JPHX on the NAFLD model induced by MCD diet. Hepatic TG and TC levels increased noticeably in the MCD vehicle group compared to those in the MCS group. JPHX 0.60 g/kg and JPHX 1.21g/kg showed significantly lower hepatic TG (Figure 2(a),* p *= 0.0007) and TC levels (Figure 2(b),* p* = 0.0003). In addition, serum levels of TG and TC in MCD-induced rats decreased, which indicated impaired secretion of TG (Figure 2(c), for week 2,* p*= 0.0004, for week 4,* p*= 0.0019, for week 6,* p*< 0.0001, and for week 8,* p* = 0.6152) and TC from hepatocytes (Figure 2(d), for week 2,* p* = 0.0002, for week 4,* p*< 0.0001, for week 6,* p *= 0.0960, and for week 8,* p*= 0.0011). These findings correlated well with the H&E and Oil red O staining results (Figure 2(e)), where the MCD diets featured macrovesicular steatosis with large droplets of fat that push the nucleus aside. Animals treated with JPHX showed lower hepatic steatosis levels than those of the MCD diet vehicle rats. Similarly, lipid droplets were detected by Oil red O staining. JPHX in the different treatment groups, but especially at 1.21g/kg, noticeably attenuated the accumulation of lipid droplets.

### 3.3. JPHX Protects Rats from MCD Diet-Induced Liver Injury and Inflammation

**Figure 3:**
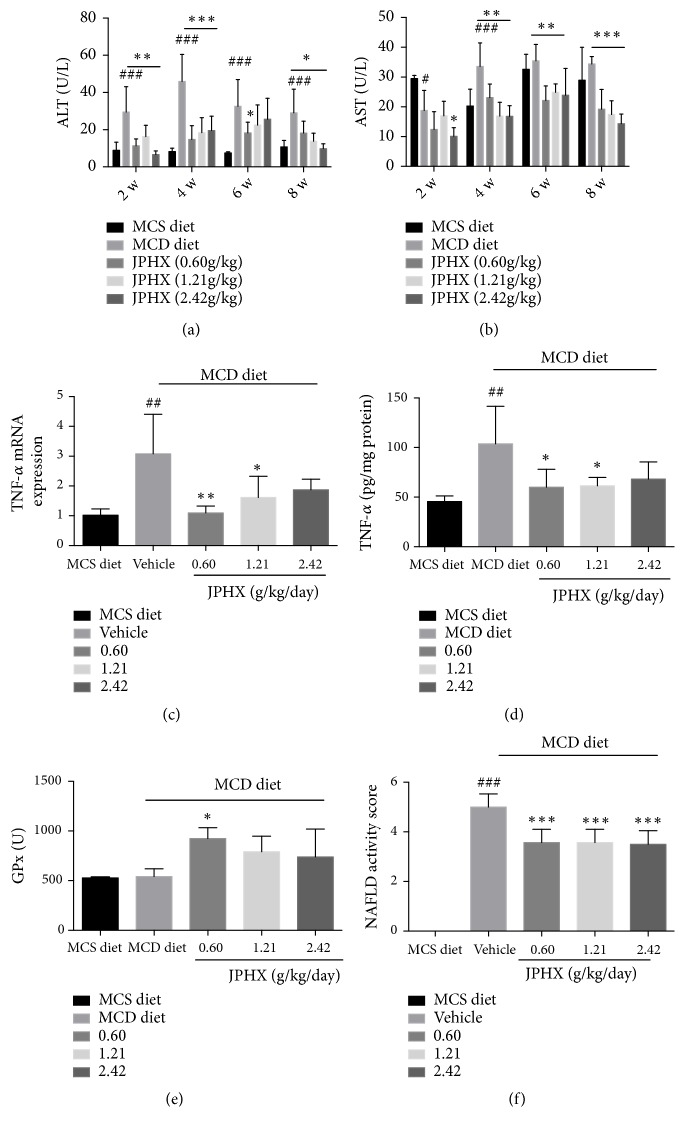
Liver injury-related parameters, inflammation-related parameters, and NAS score. (a) Serum ALT levels in rats fed with MCD or MCS diet at weeks 2, 4, 6, and 8. (b) Serum AST levels. (c) TNF-*α* mRNA expression levels were detected by qRT-PCR; data were normalized to *β*-actin. (d) Hepatic TNF-*α* concentrations were measured by an ELISA kit. (e) Effects of JPHX formula on liver GPx in rats. (f) NAFLD activity scores were analyzed by a pathologist in a blind manner. Values are shown as the mean ± SD. MCS n=6, MCD n=9, and JPHX treatment groups n=7. #*p*<0.05, ##*p*<0.01, and ###*p*<0.001, MCD diet vehicle group versus MCS diet group. *∗p*<0.05, *∗∗p*<0.01, and *∗∗∗p*<0.001, MCD diet vehicle group versus JPHX treatment groups.

Rats fed with MCD diets for 8 weeks developed NAFLD, and liver injury-related markers were detected. Serum ALT levels were dramatically increased in the MCD vehicle group due to higher ALT release from damaged liver cells. Abnormal serum ALT activities were significantly decreased by JPHX in rats (Figure 3(a), for weeks 2, 4, 6, and 8,* p*< 0.0001). Similarly, JPHX treatment reduced circulating AST level in rats compared to the MCD group (Figure 3(b), for weeks 2, 4, and 8,* p*< 0.0001; for week 6,* p*=0.0002). TNF-*α* was involved in the pathogenesis of NAFLD. MCD diets markedly increased hepatic TNF-*α* concentrations. Treatment with JPHX (0.60g/kg and 1.21g/kg) significantly decreased this MCD diet-induced elevation in TNF-*α* level (Figure 3(c),* p*=0.0059, Figure 3(d),* p* = 0.0076). These findings were confirmed by H&E, which showed decreased lobular inflammation. Oxidative stress plays an important role in the pathogenesis of NAFLD, which causes the infiltration of Kupffer cells, cell death, and liver damage [23, 24]. Reactive oxygen species (ROS) can react with the accumulated lipids in the liver to cause lipid peroxidation. Detoxifying enzymes which scavenge ROS act as the first line of defense against ROS [15, 25]. JPHX treatment group had higher expressions of liver GPx levels compared with MCD group; however only low dose of JPHX formula show significantly higher expressions of liver GPx levels (Figure 3(e), p=0.0110). Histological examination indicated that JPHX normalized the NAS score (Figure 3(f),* p*< 0.0001). Our data suggested that JPHX attenuated hepatocellular injury and inflammation, as indicated by reversal of abnormal serum ALT and AST levels and reduced TNF-*α* expression and inflammatory infiltrates.

### 3.4. JPHX Prevents Fibrosis by Inhibiting Expression of Collagen I and Matrix Metalloproteinase

**Figure 4:**
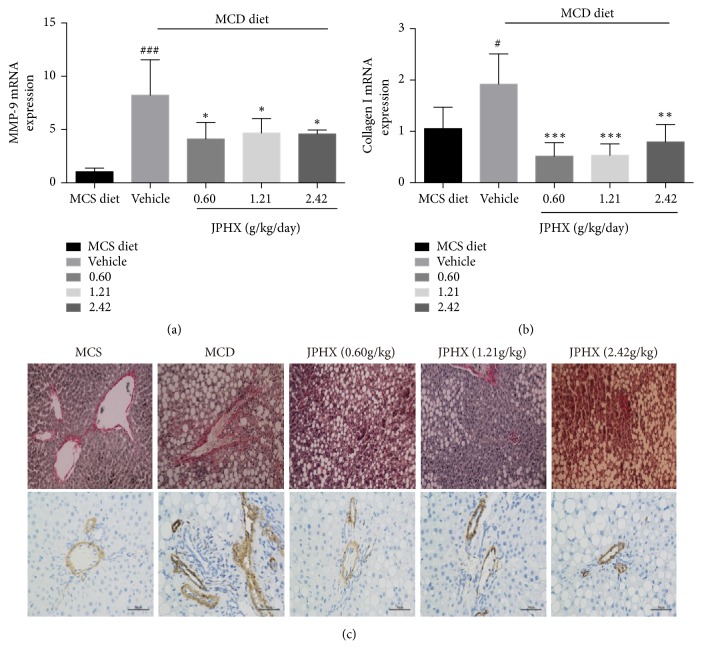
JPHX inhibits hepatic fibrosis in MCD diet-fed rats, as suggested by profibrotic gene expression level, Sirius red staining, and *α*-SMA Expressions level. (a) Collagen I and MMP-9 mRNA expression levels were detected by qRT-PCR. The relative amounts of mRNA were normalized to *β*-actin. (b) Sirius red staining showed noticeable pericellular fibrosis in the MCD group. Representative liver sections from different groups. Original magnification × 200. (c) Paraffin-embedded sections were immunostained with an *α*-SMA antibody. Representative liver sections from different groups. Original magnification × 400. Values are shown as mean ± SD. MCS n=6, MCD n=9, and JPHX treatment groups n=7. #*p*<0.05, ##*p*<0.01, and ###*p*<0.001, MCD diet vehicle group versus MCS diet group. *∗p*<0.05, *∗∗p*<0.01, and *∗∗∗p*<0.001, MCD diet vehicle group versus JPHX treatment groups.

The hepatic mRNA levels of matrix metalloproteinase-9 (MMP-9) markedly increased in the rats fed the MCD diet alone but were downregulated by low, medium, and high doses of JPHX formula (by 50.6%, 43.0%, and 44.3%, respectively). Also, collagen I mRNA, to a significantly lesser extent in the JPHX group, showed the same trend. JPHX inhibited liver fibrosis by inhibiting aberrant collagen I and MMP-9 gene expression induced by the MCD diet (Figure 4(a), for MMP-9,* p*=0.0009; for collagen I,* p*=0.0007). These findings were consistent with the Sirius red staining results. Administration of the MCD diet was associated with pericellular fibrosis, and this response was inhibited by JPHX treatment (Figure 4(b)). Hepatic stellate as the major cell type involved in liver fibrosis follows its transdifferentiation into fibrogenic myofibroblasts, which cause liver fibrosis [15]. Alpha-smooth muscle actin (*α*-SMA) is commonly used as a marker of myofibroblast formation. Immunohistochemistry analysis revealed that the expression of *α*-SMA was very weak in the MCS group, whereas it was dominant in MCD group. JPHX treatment attenuated the increased expression of *α*-SMA in the fibrotic liver (Figure 4(c)), which is consistent with the profibrotic gene expression and Sirius red staining results.

### 3.5. JPHX Formula Reduces Hepatocyte Apoptosis

**Figure 5:**
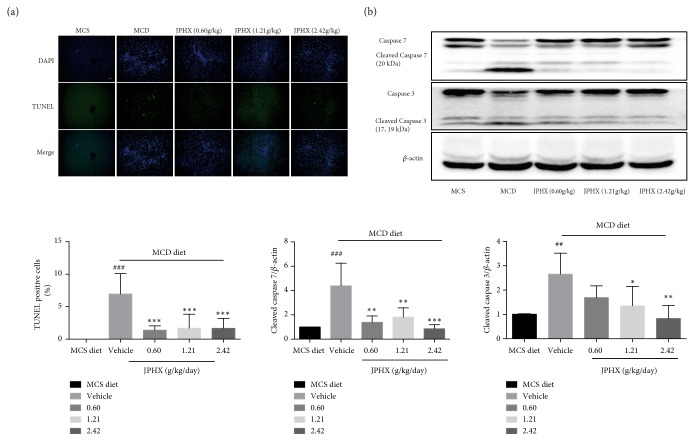
Analysis of apoptosis in liver sections and effect of JPHX on the expression levels of cleaved forms of caspase 3 and caspase 7. (a) Representative images showed apoptotic cells in the liver of different groups of rats by TUNEL staining and quantification of TUNEL-positive cells. Apoptotic nuclei stained by TUNEL (green) and counterstained with DAPI (blue) to mark nuclei; hepatocytes were imaged by confocal microscope. (b) Representative Western blotting results of total and cleaved forms of caspase 3 and caspase 7. Protein expression levels were normalized to that of *β*-actin. Values are shown as mean ± SD. MCS n=6, MCD n=9, and JPHX treatment groups n=7. #*p*<0.05, ##*p*<0.01, and ###*p*<0.001, MCD diet vehicle group versus MCS diet group.*∗p*<0.05, *∗∗p*<0.01, and *∗∗∗p*<0.001, MCD diet vehicle group versus JPHX treatment groups.

Hepatocyte apoptosis was measured by TUNEL staining and with reference to the expression levels of caspase 3 and 7 proteins. The slides showed few TUNEL-positive cells in the MCS group. The number of apoptotic hepatocytes obviously increased in the MCD diet-fed rats compared to the MCS diet group (Figure 5(a),* p*< 0.0001). Moreover, Western blotting showed that cleaved forms of caspase 3 and caspase 7 were increased in the livers of MCD diet-fed rats (Figure 5(b), for caspase 3,* p*=0.0062, for caspase 7,* p*=0.0005). We observed a significant decrease in TUNEL-positive cells, as well as cleaved caspase 3 and 7, in the JPHX treatment group compared to MCD diet feeding alone.

### 3.6. JPHX Decreases the Phosphorylation of JNK Signaling

**Figure 6:**
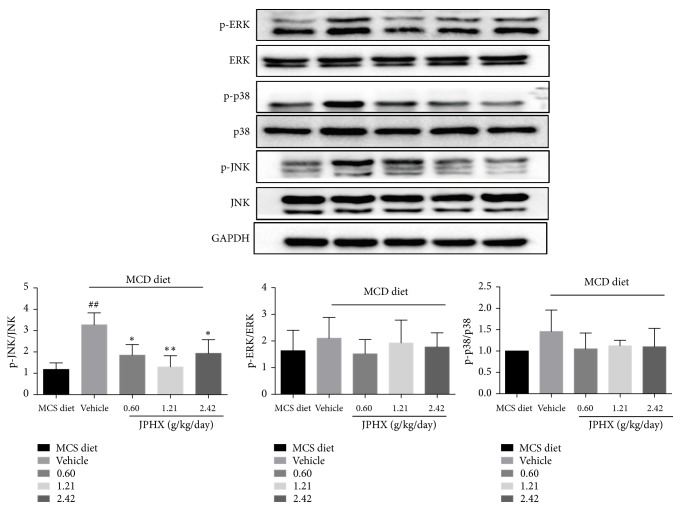
Effect of JPHX on MAPK pathway. JNK, p38, and ERK were measured from total and phosphorylated proteins. Protein expression levels were normalized to that of GAPDH. Values are showed as mean ± SD. MCS n=6, MCD n=9, and JPHX treatment groups n=7. #*p*<0.05, ##*p*<0.01, and ###*p*<0.001, MCD diet vehicle group versus MCS diet group. *∗p*<0.05, *∗∗p*<0.01, and *∗∗∗p*<0.001, MCD diet vehicle group versus JPHX treatment groups.

It is important to note that the mitogen-activated protein kinase (MAPK) pathway plays crucial roles in inflammation and apoptosis. Our data showed that JPHX prevented the NAFLD model rats from experiencing inflammation and apoptosis. Based on our data, we measured the levels of total and phosphorylated stress-activated protein kinases (SAPK)/c-Jun N-terminal kinase (JNK), p38 MAPK, and MAPK/ERK. JPHX only inhibited the hepatic protein expression of phosphorylated JNK in the liver of NAFLD rats. Moreover, treatment with JPHX did not significantly affect the expression of p-p38 and p-ERK compared with that in rats fed the MCD diet (Figure 6, for p-JNK,* p*=0.0041, for p-p38,* p*=0.1889, and for p-ERK,* p*=0.5983).

## 4. Discussion

NAFLD and its complications contributed to high liver-related and overall mortality. Currently, controlling risk factors such as obesity, diabetes, and hyperlipidemia and weight loss through physical activity and nutritional interventions are the therapeutic options of choice for the management of NAFLD and NASH [26, 27]. At present, there are no clinically available effective drugs for the management of NAFLD and NASH, although a few compounds, such as Pioglitazone and vitamin E, show promising effects for patients with NAFLD [28]. Here, our data demonstrated that JPHX treatment prevents steatosis, liver injury, inflammation, fibrosis, and apoptosis in the MCD diet-induced NAFLD model. Based on our data, JPHX may have potential as a therapeutic formula for the alleviation of NAFLD.

Since the pathogenesis of NAFLD is complex, strategies for treating NAFLD should target multiple or prominent pathological pathways. Traditional Chinese medicine (TCM), containing a cocktail of multiple bioactive components, is an alternative solution for management of multifactorial chronic diseases, like NAFLD. The protective effect of JPHX against NAFLD probably occurs through multiple anti-inflammatory and antifibrotic candidate compounds present in JPHX formula. Some studies have demonstrated that* Atractylodes macrocephal* has an anti-inflammatory effect and could regulate lipid metabolism and improve liver and kidney function [29, 30].* Alisol A 24-acetate (AA*), isolated from* Rhizoma Alismatis*, also ameliorated lipid accumulation and inflammation in the NASH model [31, 32]. Our findings suggested that JPHX attenuates the accumulation of hepatic TG and TC.* Gomishi *is the dried fruit of* Fructus Schisandra chinensis*, which demonstrated anti-inflammatory properties by suppressing inducible nitric oxide synthase in rat hepatocytes [33]. In our study, liver inflammation was attenuated, as indicated by reduced infiltration of Kupffer cells and neutrophils. We also found that increased TNF-*α* production in MCD diet-induced NASH was decreased in JPHX-treated rats. Kupffer cells produce proinflammatory cytokines like TNF-*α*, which activates HSC leading to development of the disease [34]. As the main component of JPHX prescription,* Danshen *has been widely used based on its antioxidant and anti-inflammatory properties. Recent studies showed that* Danshen *protects against early stage alcoholic liver disease in mice and its lipophilic compound, Tan IIA, inhibits lipopolysaccharide (LPS)-induced hepatic stellate cell (HSC) activation [35, 36]. The activation of HSC produces collagen, which contributes to liver fibrosis. Although fibrosis is not a component of NAS, it is strongly related to the diagnosis and prognosis of diseases and indicates a progressive liver disease [37]. Importantly, fibrosis is associated with high liver-related morbidity and mortality in humans. We demonstrated that liver fibrosis was attenuated, as shown by decreased pericellular and perisinusoidal collagen deposition and the profibrotic genes MMP-9 and Collagen I. Multiple active compounds in JPHX may interact with other compounds, resulting in the hepatoprotective effect in the experimental model of NAFLD through regulating lipid accumulation, inflammation, and fibrosis.

A variety of mechanisms contributed to the development of NAFLD. A “ two-hit” model was proposed by Day and James [38]. Lipid accumulation plays a pivotal role in liver injury. Proinflammatory cytokines and oxidative stress also contribute to the progression of NAFLD [39]. JNK is involved in both of these processes [40]. It is important to note that MAPK signaling pathway is involved in physical and chemical stress, which plays a role in liver injury [41]. Some inflammatory cytokines, such as IL-6 and TNF-*α*, activated the phosphorylation of JNK. Sustained activation of JNK contributed to cell death. Interestingly, our data showed that JNK1 phosphorylation was significantly decreased by JPHX, whereas p38 and ERK remained unaffected. The data presented herein are in agreement with a study in which the inhibition of JNK1 was an effective treatment for NAFLD [42]. Improvement of apoptosis plays a vital role in the treatment of NAFLD [43].

Most of drugs show classical sigmoidal dose-response curves which illustrate an increase in inhibitory or stimulatory effect with rising drug concentration [44]. However, in our experiments, compared with the low dose and high dose, the effects of medium-dose (1.21 g/kg) JPHX formula were more effective. This “bell-shaped” like curves could be attributed to the characteristic of TCM that the efficacy of TCM usually comes from synergistic interactions of multiple ingredients [45]. TCM treats diseases based on a holistic rule that uses herbs or formula containing multiple compounds to rebalance the organism [46]. There are multiple active compounds in JPHX formula which targets multiple pathological pathways. Multiple herbal ingredients and their targets built a complex network with rats. As a result, the efficacy of TCM usually does not follow the typical dose-response curves [47]. In the future, with the development of systems biology and pharmacology and network pharmacology, based on our study, we can use systematic docking, herb-target network analysis, and some TCM databases to go further with the JPHX formula [46, 48, 49].

Although the MCD diet has been used for over 40 years to induce NAFLD, which is similar to hepatic histological features of human NAFLD, there are still some limitations in this model [15]. NAFLD is closely related to obesity and metabolic syndrome; however, the MCD diet model of NAFLD causes weight loss and does not exhibit insulin resistance [50, 51]. There is no ideal small animal model of NAFLD to perfectly mimic the human pathology, which reflects the imperfect understanding of human complexity pathogenesis. However, some refined animal models such as genetic models and combination models of genetic and nutritional factors have been developed to verify the hypotheses on the pathogenesis of NAFLD [52, 53]. In the future, further researches on animal model will develop novel diagnostic and therapeutic approaches to help people with NAFLD.

## 5. Conclusion

In conclusion, the MCD experimental model animals developed severe livery injuries, including steatosis, inflammation, and fibrosis within 8 weeks in our study. JPHX exhibited hepatoprotective effects against NAFLD by targeting lipid accumulation, fibrosis, inflammation, and apoptosis. These findings suggest that JPHX may be a promising therapeutic formula for the treatment of NAFLD.

## Figures and Tables

**Table 1:** Primers information.

Target genes	Primers	Sequence
TNF*α*	Forward Primer	GCCCAGACCCTCACACTC
	Reverse Primer	CCACTCCAGCTGCTCCTCT
Collagen I	Forward Primer	AGGCATAAAGGGTCATCGTG
	Reverse Primer	ACCGTTGAGTCCATCTTTGC
MMP-9	Forward Primer	GACAATCCTTGCAATGTGGATG
	Reverse Primer	CCGACCGTCCTTGAAGAAATG
*β*-actin	Forward Primer	AGCCATGTACGTAGCCATCC
	Reverse Primer	CTCTCAGCTGTGGTGGTGAA

## Data Availability

The data used to support the findings of this study are available from the corresponding author upon request.
